# Screw Stimulation Thresholds for Neuromonitoring in Minimally Invasive Oblique Lateral Lumbar Interbody Fusion (OLLIF): A Correlational Study

**DOI:** 10.7759/cureus.62859

**Published:** 2024-06-21

**Authors:** Hamid Abbasi, Dominic J Moore, Mohadese Rajaeirad, Jiawen Zhan

**Affiliations:** 1 Spine Surgery, Inspired Spine Health, Minneapolis, USA; 2 Biomechanics, University of Isfahan, Isfahan, IRN; 3 Machine Learning, Inspired Spine Health, Minneapolis, USA

**Keywords:** minimally invasive surgical procedures, minimally-invasive spine, lumbar-fusion, intra-operative neuromonitoring, spine surgury

## Abstract

Introduction: This study presents findings from an investigation into the correlation of neuromonitoring techniques in minimally invasive lumbar fusions and their open counterparts regarding acceptable thresholds for screw stimulation. The threshold for acceptable stimulation value for open surgery has been established. The study compared acceptable thresholds for open pedicle screws where there is more connection between the screw and the soft tissue.

Methods: The neuromonitoring data of 17 patients who underwent oblique lateral lumbar interbody fusion (OLLIF) procedures between September 2023 to May 2024 were reviewed. Neuromonitoring was conducted throughout surgeries, recording stimulation thresholds for pedicle screws insulated and uninsulated, to simulate the environment of a screw during open and minimally invasive surgery respectively. Patients' BMI was also collected for potential correlation analysis.

Results: Results indicate a discernible correlation between stimulation thresholds in open and minimally invasive surgeries, but no definitive correlation with BMI due to sample size limitations. Though a significant correlation between the two stimulating styles is apparent, there is a good correlation to suggest what threshold should determine a standard stimulation threshold for minimally invasive surgeries.

Conclusion: The study emphasizes the need for refined neuromonitoring strategies in minimally invasive spinal fusion (MISF) surgeries to ensure patient safety and surgical effectiveness. Further research with larger cohorts is recommended to establish optimized protocols that have a clearly defined amplitude for MISF thresholds.

## Introduction

Spinal surgeries, particularly those involving lumbar fusion, are intricate procedures that require careful navigation around neural structures to prevent post-operative complications. In many of these surgeries, stabilization is performed by placing pedicle screws in levels above and below the discs that are fused, stimulating the screws after they are placed is gaining more popularity and is becoming a standard of care. Over the last few decades, neuromonitoring techniques have become indispensable in spinal surgeries, first in cases with extensive correction, such as scoliosis, and now even in the most minimally invasive of procedures [1]. The process of neuromonitoring aids surgeons in real-time assessment of neurologic pathways to prevent inadvertent nerve damage using modalities like somatosensory evoked potentials (SSEP), electromyography (EMG), and motor evoked potentials (MEP) [2]. Through these modalities and the use of stimulating probes, surgeons can gauge the proximity of nerves to surgical instruments and hardware by observing the amplitude required to elicit a response in neuromonitoring sensors [3]. More specifically, any breaching of the pedicle cortex can be assessed by eliciting activity from muscle groups that correspond to nearby nerve roots, and subsequently, the screw can be repositioned accordingly [4].

In traditional open spinal surgeries, the pathway for neuromonitoring stimulation is relatively straightforward, as the signal travels through hardware, bone, and tissue to reach the nerves. Consequently, surgeons can often obtain clear indications of nerve proximity based on the observed amplitudes. Though typical amplitudes used for a stimulation threshold are not universally agreed upon, amplitudes of 7-8mA are generally accepted, with any response below this value indicating that the screw is too close to any adjacent nerve or has breached the insulating cortical bone [4-6]. More specifically, the threshold of 6 milliamps or below is considered a breach of the pedicle screw, between 6 and 8 is considered a possible breach, above 8 is generally considered safe, and above 12 it is rare that the pedicle screw has contact with neural structure [7,8]. In open surgery, the screw is directly placed into the bone with no significant contact with the surrounding soft tissue and minimal energy dispersion, while in minimally invasive surgery (MIS), a pedicle screw is placed and a significant portion of the screw has contact with the soft tissue, dissipating energy of the stimulation probe [9-11]. This interference results in an increase in the amplitude measured to obtain a clear and actionable response as opposed to its traditional open counterpart. Not enough data is available to understand how much of this energy is dissipated and how energy reaches the tip of the screw. We do not have enough data to correlate and compare MIS to open surgeries to understand the safe threshold for MIS, and this study aims to change that.

Understanding the nuances of neuromonitoring amplitudes in minimally invasive lumbar fusion surgeries is paramount for optimizing patient outcomes and minimizing the risk of nerve injury [12,13]. The present study seeks to bridge the knowledge gap by investigating the correlation between the amplitudes required in minimally invasive procedures in comparison to those in traditional open surgeries [10,14]. By simulating the environment of open surgeries through a process of insulation, our research's primary objective is to discern corresponding amplitude thresholds that would signify a similar correlation, thereby enhancing the surgeon's ability to prevent neurological complications during minimally invasive spinal fusion (MISF) [15]. In this case, any possible contributions that body mass index (BMI) has to thresholds will be analyzed as a secondary objective. The outcomes of this research have the potential to significantly improve intraoperative neuromonitoring strategies, resulting in safer surgical procedures and better patient outcomes.

## Materials and methods

Study design

This study is a correlational study that reviews neuromonitoring data from 17 patients who underwent spinal fusion via trans-Kambin oblique lateral-posterior interbody fusion (OLLIF) from September 11th, 2023, to May 6th, 2024. All surgical procedures were performed by a single board-certified spine surgeon who worked across four different medical facilities, hospitals, and surgery centers in Minnesota and Kansas, United States. This allowed for a consistent surgical approach and neuromonitoring protocol to be established and adhered to during the study period.

Patient selection

The Institutional Review Board (IRB) exemption for this study was sought and granted by Pearl Pathways IRB. This exemption facilitated the retrospective review of patient data without requiring additional consent beyond the treatment agreements in place for the OLLIF procedures. Inclusion criteria for this study were patients above the age of 18 who would undergo MISF with OLLIF surgery accompanied by intraoperative imaging and intraoperative neuromonitoring, who then during surgery noted an intraoperative stimulation value below 20mA. The exclusion criteria were straightforward, any patient records that failed to meet the specified inclusion criteria were not considered for data analysis in this study.

Procedure

In order to obtain the aforementioned data, the OLLIF procedure was conducted as a minimally invasive technique intended for spinal fusion, wherein access to the intervertebral disc space was achieved obliquely to avoid disruption of the major anterior vessels and posterior muscular structures. The surgery involved a tubular access system to aid in minimizing tissue trauma and enhancing patient recovery post-surgery then posterolateral instrumentation and fusion were performed by placing bilateral percutaneous cannulated pedicle screws [16].

Intraoperative fluoroscopy was used to verify the correct trajectory and placement of surgical screws during the procedure. In addition to being required for data collection, concurrent neuromonitoring, conducted by board-certified technologists utilizing standardized protocols, was implemented as a safety measure designed to monitor and protect neural structures during the procedure. The neuromonitoring data parameters were calibrated and interpreted in the context of each patient's baseline measurements, taken immediately following the induction of anesthesia. This real-time monitoring consisted of SSEPs, EMG, and MEPs to detect any changes in neural pathway conduction or nerve integrity, allowing immediate corrective actions during the operation.

**Figure 1:**
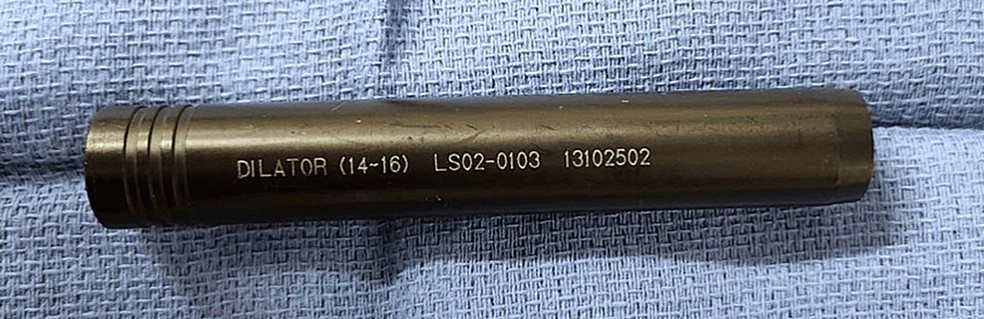
The image shows the plastic insulating tube used during the study. The design, based loosely on dilators used, allows for reduced shunting of signals to surrounding tissue.

**Figure 2:**
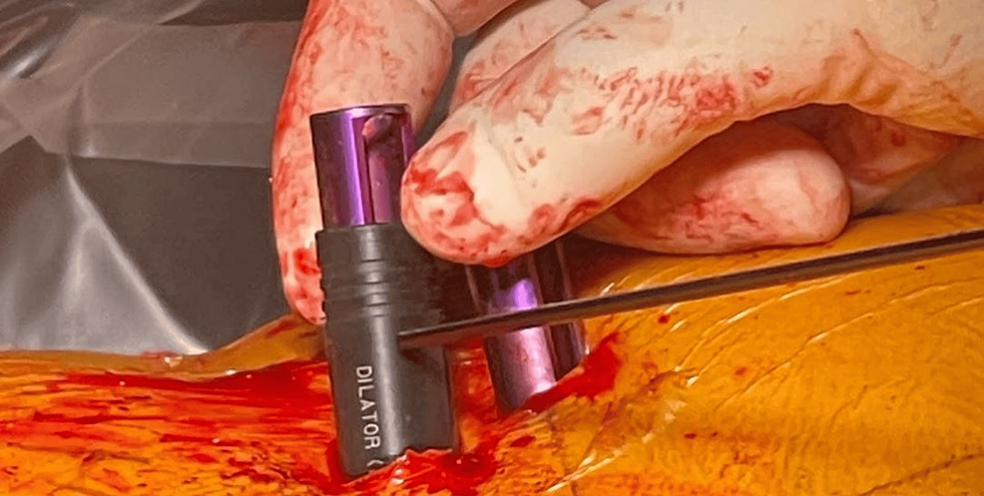
The insulating tube described in Figure 1 is shown here intraoperatively, positioned around the left L5 screw after placement to appropriately sheath it.

**Figure 3:**
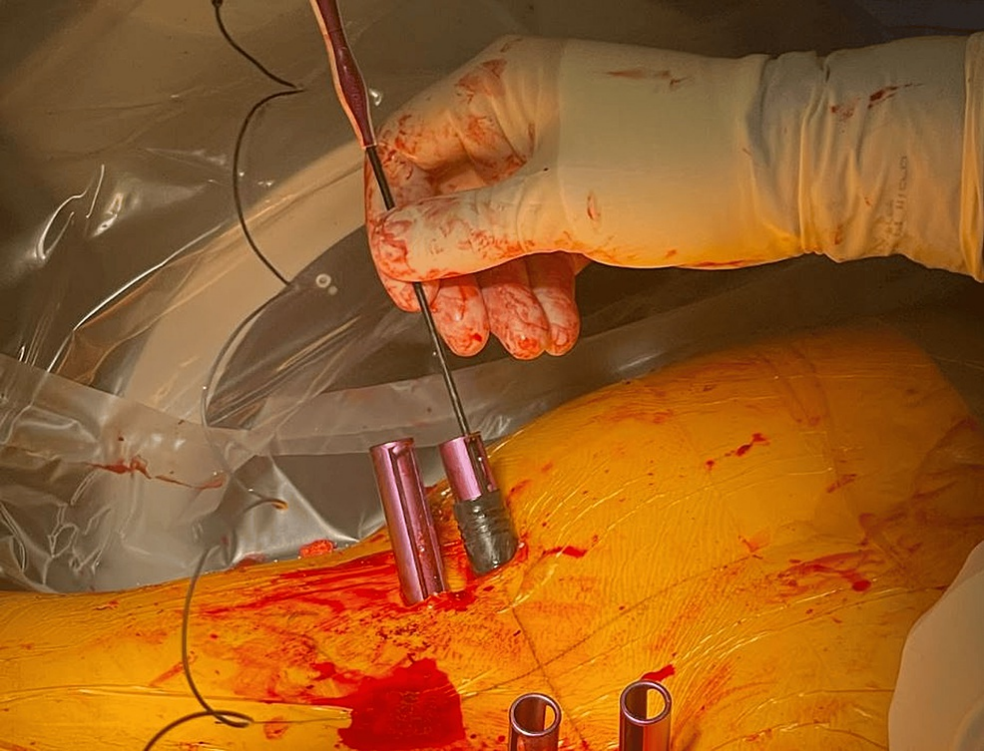
Intraoperative neurostimulation of the right S1 screw to threshold while it was sheathed in the insulating tube.

Following the surgical placement of the screws at their desired depths, neuromonitoring proceeded with the collection of study data. The screws were stimulated using a neuromonitoring probe to elicit responses, and the resulting amplitude was carefully recorded. In cases the pedicle screw returned stimulation results below 20 mA, the screw was selected to undergo further investigation. Each screw then underwent a subsequent stimulation after being encased in a plastic insulation tube and reentering it for neurophysiology equal to an open screw (as shown in Figures 1, 2, 3) whereupon the elicited amplitudes were again documented for comparison.

## Results

**Figure 4:**
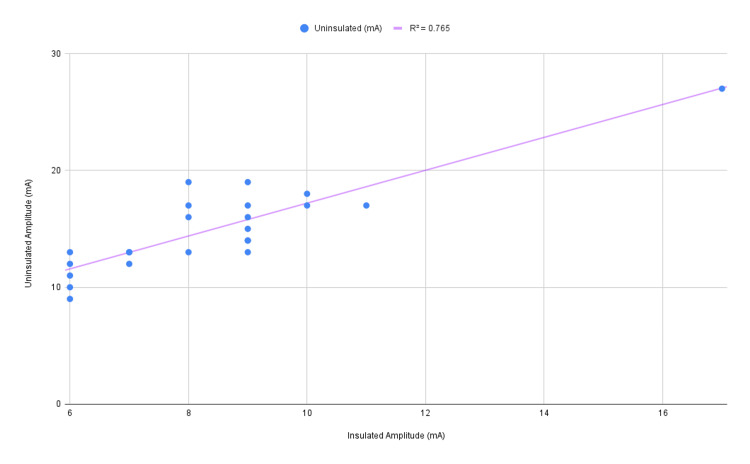
Graphed relationship of insulated and uninsulated thresholds in mA

The purpose of this report is to present the results of the correlation analysis conducted on the study variables that show a strong positive correlation between thresholds. This was done using data collected and prepared in Statistical Package for the Social Sciences (IBM SPSS Statistics for Windows, IBM Corp., Version 27.0, Armonk, NY). The study aimed to investigate potential correlations between insulated and uninsulated thresholds, as well as their associations with BMI, which are recorded in Table 1 and graphed in Figure 4 with a coefficient of determination equal to 0.765.

**Table 1:** Recorded thresholds and BMI BMI: body mass index

Insulated (mA)	Uninsulated (mA)	BMI
6	10	22.5
7	13	
8	16	32.6
6	11	
7	12	
9	19	22.6
6	13	26.3
9	13	21.9
17	27	26.3
11	17	
6	9	30.2
10	17	
9	14	27.4
8	13	22.2
9	15	26.8
7	13	
10	18	22.9
9	14	37.2
8	19	28.5
9	17	43.4
9	16	41.3
8	17	33.9

Data was imported into SPSS, cleaned, and prepared for analysis. Pearson's correlation coefficient was chosen as the appropriate method due to its suitability for assessing linear relationships between continuous variables, considering the interval scale of measurement for the variables and the sample size (N=23). Table 2 displays the results of the correlation analysis.

**Table 2:** Pearson correlation's output ** Correlation is significant at the 0.01 level (2-tailed).

Correlations		Insulated Threshold (mA)	Uninsulated Threshold (mA)	BMI
Insulated Threshold (mA)	Pearson Correlation	1	0.875^**^	-0.023
	Sig. (2-tailed)		0.000	0.917
	N	23	23	23
Uninsulated Threshold (mA)	Pearson Correlation	0.875^**^	1	0.037
	Sig. (2-tailed)	0.000		0.867
	N	23	23	23
BMI	Pearson Correlation	-0.023	0.037	1
	Sig. (2-tailed)	0.917	0.867	
	N	23	23	23

## Discussion

The analysis revealed a statistically significant positive correlation between insulated and uninsulated thresholds (r = 0.875, p < 0.01), indicating a strong linear relationship between these variables with a large effect size. However, no statistically significant correlations were found between either insulated or uninsulated thresholds and BMI. These findings suggest that BMI may not be significantly associated with the thresholds measured in this study.

The goal of this study was to elucidate whether a correlation exists between the neural monitoring stimulation thresholds for pedicle screws during MISF procedures and those employed in traditional open spinal surgeries. In the context of our research, we utilized a cohort of 17 patients undergoing OLLIF procedures and meticulously recorded neural monitoring stimulation thresholds, both uninsulated and subsequently insulated using a plastic insulating tube. Our findings indicate that there is indeed a discernible relationship between uninsulated and insulated stimulation thresholds. However, the data at hand does not permit us to construct a precise predictive model or equation to describe this correlation conclusively, nor establish a definitive statistical standard for practical application during MISF interventions.

In tandem with stimulation threshold relationships, our study also investigated the potential correlation between BMI and neural monitoring stimulation amplitudes. Studies have long shown that surgical interventions in the lumbar spine are met with a significant increase in complications due to BMI ≥35, and as such, surgeries like the OLLIF, which do not show significant effects of BMI on perioperative outcomes, will likely become far more common for this category of patient [17,18]. Therefore, the relationship between BMI and amplitude in these deserves to be assessed, yet the relationship between these variables remains elusive, with no definitive correlation observed. This inconclusiveness could be attributable to the limited size of the data pool, which does not yet provide adequate statistical power to support or refute a correlation. It is imperative to underscore the preliminary nature of this research, which, to our knowledge, represents the first endeavor to formulate a benchmark for neural monitoring thresholds specific to MISF application with regard to screw stimulation.

Past research has successfully delineated general safety thresholds for neuromonitoring in the realm of traditional spinal procedures, emphasizing the significance and utility of our research [19]. At present, this research suggests that further data collection would be able to yield analogous threshold standards for MISF, thereby enhancing procedural safety and outcomes. This would allow for safer MISF surgeries due to this established standard, thereby yielding improved clinical outcomes by reducing nerve irritation or possible damage.

Notwithstanding our promising findings, we must recognize the limitations inherent in our current study, as mentioned previously. The sparsity of data points and the restricted sample size have likely played a role in tempering the strength and clarity of the observed correlations. Prospective research endeavors should prioritize an expansion of the sample size to mitigate these limitations.

It is within this framework that we propose the extension of our investigation to further delineate the correlation between neuromonitoring stimulation thresholds and the affixed variables, both in MISFs, such as OLLIF, and in comparison to traditional open techniques. Additionally, future studies should aim to definitively determine the impact of BMI on neuromonitoring variability, thereby extending the scope of neuromonitoring applicability in spinal surgery.

## Conclusions

This proof of concept study represented a pioneering investigation into the correlation of neural monitoring screw stimulation thresholds in MISF procedures, such as OLLIFs, compared to traditional open spinal surgeries. Despite the limitations posed by the scale of the patient population and dataset, there is a discernible correlation between the screw stimulation thresholds of insulated and uninsulated environments. With further research and a larger cohort, this correlation may be further defined to elucidate a precise standard by which MISF techniques might evaluate their intraoperative neuromonitoring, as well as provide conclusive evidence for or against a definitive relationship between BMI and neural monitoring thresholds.

Irrespective of the inconclusive findings regarding BMI, the outcomes of this study carry significant weight for the development of more effective neuromonitoring strategies in the realm of minimally invasive spinal surgery. They serve as an essential foundation for future research, which will be critical to further elucidate the nuances of neuromonitoring in this context and establish optimized standards aimed at maximizing patient safety and surgical efficacy for the growing field of minimally invasive spine surgery.
